# Pressure overload-induced mild cardiac hypertrophy reduces left ventricular transmural differences in mitochondrial respiratory chain activity and increases oxidative stress

**DOI:** 10.3389/fphys.2012.00332

**Published:** 2012-08-28

**Authors:** Michel Kindo, Sébastien Gerelli, Jamal Bouitbir, Anne-Laure Charles, Joffrey Zoll, Tam Hoang Minh, Laurent Monassier, Fabrice Favret, François Piquard, Bernard Geny

**Affiliations:** ^1^Service de Chirurgie Cardiovasculaire, Pôle d'activité médico-chirurgicale Cardiovasculaire, Hôpitaux Universitaires, CHRU StrasbourgStrasbourg, France; ^2^Equipe d'Accueil 3072, Faculté de Médecine, Institut de Physiologie, Université de StrasbourgStrasbourg, France; ^3^Service de Physiologie et d'Explorations Fonctionnelles, Pôle de Pathologie Thoracique, Hôpitaux Universitaires, CHRU StrasbourgStrasbourg, France; ^4^Laboratoire de Neurobiologie et de Pharmacologie, Université de StrasbourgStrasbourg, France; ^5^Faculté des Sciences du Sport, Université de StrasbourgStrasbourg, France

**Keywords:** aortic banding, pressure overload, heart, hypertrophy, mitochondria, oxydative stress, transmural

## Abstract

**Objective:** Increased mechanical stress and contractility characterizes normal left ventricular (LV) subendocardium (Endo) but whether Endo mitochondrial respiratory chain complex activities is reduced as compared to subepicardium (Epi) and whether pressure overload-induced LV hypertrophy (LVH) might modulate transmural gradients through increased reactive oxygen species (ROS) production is unknown. **Methods:** LVH was induced by 6 weeks abdominal aortic banding and cardiac structure and function were determined with echocardiography and catheterization in sham-operated and LVH rats (*n* = 10 for each group). Mitochondrial respiration rates, coupling, content and ROS production were measured in LV Endo and Epi, using saponin-permeabilized fibers, Amplex Red fluorescence and citrate synthase activity. **Results:** In sham, a transmural respiratory gradient was observed with decreases in endo maximal oxidative capacity (−36.7%, *P* < 0.01) and complex IV activity (−57.4%, *P* < 0.05). Mitochondrial hydrogen peroxide (H_2_O_2_) production was similar in both LV layers. Aortic banding induced mild LVH (+31.7% LV mass), associated with normal LV fractional shortening and end diastolic pressure. LVH reduced maximal oxidative capacity (−23.6 and −33.3%), increased mitochondrial H_2_O_2_ production (+86.9 and +73.1%), free radical leak (+27.2% and +36.3%) and citrate synthase activity (+27.2% and +36.3%) in Endo and Epi, respectively. Transmural mitochondrial respiratory chain complex IV activity was reduced in LVH (−57.4 vs. −12.2%; *P* = 0.02). **Conclusions:** Endo mitochondrial respiratory chain complexes activities are reduced compared to LV Epi. Mild LVH impairs mitochondrial oxidative capacity, increases oxidative stress and reduces transmural complex IV activity. Further studies will be helpful to determine whether reduced LV transmural gradient in mitochondrial respiration might be a new marker of a transition from uncomplicated toward complicated LVH.

## Introduction

Chronic pressure overload (PO) is a frequent clinical occurrence that leads to left ventricular hypertrophy (LVH). Initially, LVH was considered to be a compensatory mechanism that allows the normalization of wall mechanical stress and generation of the required cardiac output (Opie et al., 2006). However, LVH is also a risk factor for the development of heart failure and sudden death from arrhythmias. Thus, systemic hypertension and aortic stenosis-induced LVH can become maladaptive and precede the onset of heart failure (Drazner, 2011). Accordingly, recent data support that enhanced post-operative LV mass regression after aortic valve replacement for aortic stenosis is associated with improved long-term survival (Ali et al., 2011).

LVH results from increased cardiomyocyte size induced by an increase in the number of intracellular organelles, including mitochondria. Mitochondria are the main energy powerhouses of cells, and they convert nutrients into energy through cellular respiration. Although no unique pattern of cardiac adaptation occurs in response to physiological or pathological hypertrophic stimuli, mitochondrial dysfunction and increased oxidative stress are the primary factors in LVH pathogenesis (Ventura-Clapier et al., 2001; Seddon et al., 2007; Takimoto and Kass, 2007; Rimbaud et al., 2009). Cardiac metabolic flexibility is crucial and during PO-induced cardiac hypertrophy, the heart shifts from fatty acids to glucose as the primary substrate for energy production. Later, in end-stage heart failure, mitochondrial dysfunction may reduce the rate of substrate oxidation, decreasing energy production and increasing the formation of reactive oxygen species (ROS) (Ventura-Clapier et al., 2001; Zoll et al., 2005; Seddon et al., 2007; Takimoto and Kass, 2007; Ingwall, 2009). Under conditions of high oxidative stress, cell dysfunction, necrosis and/or apoptosis are induced (Seddon et al., 2007; Takimoto and Kass, 2007).

Oxidative stress is related to an excess production of ROS originating from several sources in the heart. Besides mitochondria, xanthine oxidase, uncoupled nitric oxide synthases, inflammatory cells, and NADPH oxidases are largely involved in cardiac remodeling, including during cardiac PO (Murdoch et al., 2006; Nabeebaccus et al., 2011). ROS include several free radicals like superoxide anion, hydroxyl and the highly reactive compound hydrogen peroxide (H_2_O_2_). H_2_O_2_, resulting from superoxide dismutase action on superoxide anion, is considered as the most relevant ROS signaling molecule in cells which can give rise to hydroxyls radicals and may impair mitochondrial respiration rates (Veal et al., 2007). We therefore investigated an eventual relationship between increased H_2_O_2_ production and LV subendocardium (Endo) and subepicardium (Epi) mitochondrial function.

Indeed, despite its frequency, relatively few data are available and whether impaired mitochondrial oxidative capacity occurs in mild cardiac hypertrophy is still discussed (Jameel and Zhang, 2009; Rimbaud et al., 2009; Van Bilsen et al., 2009; Griffiths et al., 2010).

Interestingly, in line with their different origins during embryologic development, the LV Endo and Epi present distinct characteristics in terms of blood flow, structure, metabolism, electrophysiological, and contractile properties (Whitty et al., 1976; Cazorla et al., 2005; Sengupta et al., 2006; Duncker and Bache, 2008; Van der Velden et al., 2011). Such transmural differences are well known but, whether they are associated with transmural differences in mitochondrial complex activities deserves further analysis. Indeed, differences in subendo- and Epi mitochondria or their regulation were expected but, previous reports showed no or minor differences when considering metabolic enzymes or electron transport system (Geshi et al., 1988; Gallego-Delgado et al., 2006). However, there are few data comparing endo- and epicardium layers mitochondrial function in the normal LV, especially using saponin-skinned fibers that ensure global mitochondrial function assessment in intact mitochondria (MacDonald et al., 2011).

The aim of this study was to challenge the hypothesis that, accordingly to the non-uniform properties of the LV wall, (1) mitochondrial respiratory chain complex activities of LV Endo are reduced as compared to Epi and (2) that increased mechanical wall stress that occurs in LVH might impair such a transmural respiratory gradient. To get insight in the mechanisms involved, we also investigated ROS production in the Endo and Epi layers through mitochondrial hydrogen peroxide measurements both in normal heart and after PO-induced LVH.

## Methods

Adult male Wistar rats (weight range 250–300 g) were used in the experiments. Animals were kept in a temperature- and humidity-controlled room with a 12:12 h photoperiod and were provided with food and water *ad libitum*. The study conformed to the *Guide for the Care and Use of Laboratory Animals* published by the US National Institutes of Health and was approved by the institutional animal care committee.

### Anaesthesia and experimental design

Twenty animals were randomly assigned to two groups of ten each. Sham-operated rats underwent a procedure similar to that applied to the LVH group except that the aorta was not banded in sham-operated animals. In the LVH group, the abdominal aorta was banded above the renal arteries. During the procedure, anaesthesia was induced with 3% isoflurane and oxygen (1 l/min) in an induction chamber (Minerve, Esternay, France). After the peritoneum was opened, 5 mg/kg of intraperitoneal tramadol was administered. Anaesthesia was maintained with 1.5% isoflurane and 1 l/min oxygen at under spontaneous ventilation. The animal's core body temperature was maintained at 37°C using a controlled heating pad (Homeothermic blanket control unit, Harvard Apparatus®). The suprarenal abdominal aorta was exposed after a laparotomy was performed. A 4-0 silk suture was used to tie off the suprarenal abdominal aorta using a blunted 22 gauge needle, which was then pulled out. Following the surgery, the animals' housing conditions were kept constant during a 6-week period.

In animal models of PO secondary to aortic banding, ventricular remodeling and function depend upon several factors including localization of the aortic banding, degree of aortic coarctation and age of the animal at the time of the surgery. Our experimental design was expected to induce a progressive LVH with no alteration of the systolic ventricular function. These parameters were chosen in order to mimic common clinical settings such as systemic hypertension or aortic stenosis.

### Echocardiography and cardiac remodelling

Two days before the animals sacrifice, echocardiography was performed. The following parameters were measured in isoflurane-anaesthetized rats by two-dimensional and Doppler echocardiography (Sonos 5500, Philips Ultrasound) with a 12-MHz phased-array transducer (S12): percentage of LV fractional shortening, LV ejection fraction, LV mass, heart rate, LV end-diastolic, and end-systolic diameter, septum wall thickness in diastole, posterior wall thickness in diastole and isovolumic relaxation time. The LV mass was derived from the cubic equation at end diastole as follows: LV mass = 0.8 (1.4) [(interventricular septal dimension at diastole + LV end-diastolic dimension + posterior wall thickness at diastole)^3^ − (LV end-diastolic dimension)^3^] + 0.6 (Seddon et al., 2007). The isovolumic relaxation time, marker of the diastolic dysfunction in correlation with the filling pressure, was measured with Doppler imaging using the apical four-chamber view (Cantor et al., 2005).

### Hemodynamic measurements and cardiac sampling

At the end of the experiments, the rats were anaesthetized as previously described, and the right carotid artery was catheterized (arterial catheter, 3Fr, Vycon) for the recording of systolic, diastolic and mean arterial pressures (PowerLab, AD Instruments). The catheter was then pushed, and the LV end-diastolic pressure (LVEDP) was measured.

Thereafter, a sternotomy was performed. The still-beating heart was rapidly harvested and rinsed in ice-cold 0.9% NaCl solution, and the heart and the LV were weighed. The LV-free wall was dissected, and Endo and Epi myocardial samples were extracted under binocular microscopy (Endo and Epi were the innermost and outermost layers of the LV-free wall, respectively). Samples were used immediately for the measurement of mitochondrial function and H_2_O_2_ production.

### Myocardial mitochondrial respiration and coupling

Mitochondrial respiration in saponin-skinned fibers was studied as previously reported and recently reviewed, ensuring determination of global mitochondrial function within the architectural environment of the muscle fiber (Veksler et al., 1987; Zoll et al., 2006; Kuznetsov et al., 2008). Briefly, fibers were separated and permeabilized in a bath of solution S containing saponin (50 μg/mL) for 30 min at 4°C with shaking. The permeabilized fibers were then washed for 10 min with shaking to remove saponin and rinsed twice with the respiratory solution for 5 min to remove any phosphates. The oxygen consumption of the fibers was measured polarographically using a Clark-type electrode in a 3-mL oxygraphic cell (Strathkelvin Instruments, Glasgow, Scotland). Basal oxygen consumption (*V*_*0*_) and maximal fiber respiration (*V*_max_) rates were measured at 22.1°C under continuous stirring in the presence of a saturating amount of adenosine diphosphate (ADP) as a phosphate acceptor.

The relative contributions of respiratory chain complexes I, III and IV to global mitochondrial respiratory rates were also determined. When *V*_max_ was recorded, the electron flow occurred in complexes I, III, and IV because of the presence of glutamate (5 mM) and malate (2 mM). Complex I was blocked with amytal (0.02 mM), and complex II was stimulated with succinate (25 mM). The measurement of mitochondrial respiration under these conditions permitted the determination of complex II, III, and IV activities (*V*_succ_). After this measurement was completed, N, N, N', N'-tetramethyl-p-phenylenediamine dihydrochloride (TMPD, 0.5 mM) and ascorbate (0.5 mM) were added as artificial electron donors to cytochrome c. Under these conditions, the activity of cytochrome c oxidase (complex IV) was increased to its maximum and could be determined as an isolated step in the respiratory chain (*V*_tmpd_). Following the completion of these measurements, the fibers were harvested and dried for 15 min at 150°C. Respiration rates are expressed as μmol O_2_/min/g dry weight.

Concerning mitochondrial coupling, when permeabilized fibers are used instead of isolated mitochondria, endogenous ATPases prevent the establishment of state 4. The “state 2” is then used. This state is the basal oxygen consumption under glutamate and malate substrates. The ratio (state 3 rate)/(state 2 rate) is called the acceptor control ratio (ACR) and is viewed as an approach of mitochondrial coupling (Duteil et al., 2010).

### H_2_O_2_ production in permeabilized fibers

The H_2_O_2_ production in response to the sequential addition of substrates and inhibitors was assessed in permeabilized Endo and Epi fibers (Anderson and Neufer, 2006). H_2_O_2_ production was measured with Amplex Red reagent (Invitrogen); this reagent reacts in 1:1 stoichiometry with H_2_O_2_ in a reaction catalyzed by HRP (horseradish peroxidase; Fluka Biochemika) to yield the fluorescent compound resorufin and a molar equivalent O_2_. Resorufin has excitation/emission wavelengths of 563/587 nm and is highly stable once formed. Fluorescence was measured continuously [change in fluorescence (ΔF)/sec] with a Fluoromax 4 (Jobin Yvon) spectrofluorometer equipped with temperature control and magnetic stirring. After the baseline in the presence of ΔF (reactants only) was established, the reaction was initiated by addition of a permeabilized fiber bundle to 600 μL of buffer Z with glutamate (5 mM) and malate (2.5 mM) as substrates for complex I and succinate (5 mM) as a substrate for complex II. The addition of ADP (2 mM) to the reaction buffer led to a reduction in H_2_O_2_ release, as expected when the electron flow through the respiratory chain is stimulated. Finally, addition of the complex I inhibitor amytal (2 mM) and the complex III inhibitor antimycin (8 μM) led to interruption of the normal electron flow and induced an increase in the H_2_O_2_ release.

### Mitochondrial free radical leak

The free radical leak correspond to the H_2_O_2_ emission *per* O_2_ flux (Picard et al., 2011). H_2_O_2_ production and O_2_ consumption were measured in parallel in the same sample under similar experimental conditions. This allowed the calculation of the fraction of electrons out of sequence which reduce O_2_ to ROS in the respiratory chain (the percent of free radical leak) instead of reaching cytochrome oxidase to reduce O_2_ to water (Anderson et al., 2009). Because two electrons are needed to reduce 1 mole of O_2_ to H_2_O_2_, whereas four electrons are transferred in the reduction of 1 mole of O_2_ to water, the percent free radical leak was calculated as the rate of H_2_O_2_ production divided by two times the rate of O_2_ consumption, and the result was multiplied by 100 (Bouitbir et al., 2012).

### Citrate synthase activity

We evaluated the global mitochondrial content in Endo and Epi layers by measuring the activity of citrate synthase (Srere, 1969). Citrate synthase activity was expressed as units of activity per gram of tissue wet weight (IU.g ww).

### Statistical analysis

Values are expressed as the mean ± standard error of the mean (SEM). Comparisons of the group means were analyzed by the Mann–Whitney test or by a Student *t*-test. *P* < 0.05 was considered to be statistically significant. Statistical analysis was performed using the Prism database (GraphPad Prism 5, Graph Pad Software Inc., San Diego, CA, USA).

## Results

### Clinical, anatomical, and echocardiographic characteristics of the sham-operated and the left ventricular hypertrophy groups

PO induced LV remodeling with significant cardiac hypertrophy, as inferred from the 31.7% increase in LV mass (Tables 1 and 2). The LV remodeling was concentric and involved similar thickening of the septal and the posterior walls (Table 2). None of the animals developed clinical evidence of heart failure or impaired systolic or diastolic cardiac functions, as inferred from the normal echocardiographic values of LV fractional shortening, ejection fraction and isovolumic relaxation time and of LV end diastolic pressures in the LVH rats.

**Table 1:** **Clinical and anatomical characteristics in sham-operated and LV hypertrophy groups**.

	**Sham-operated (*n* = 10)**	**LVH (*n* = 10)**	***P***
Heart rate (cycles/min)	395.68 ± 10.43	394.51 ± 12.43	NS
SAP (mmHg)	119.09 ± 3.91	142.46 ± 8.14	0.016
DAP (mmHg)	94.85 ± 5.32	104.59 ± 9.34	NS
MAP (mmHg)	105.3 ± 3.88	116.54 ± 9.54	NS
LVEDP (mmHg)	6.81 ± 1.10	7.77 ± 1.06	NS
Body weight (g)	500.30 ± 11.31	477.80 ± 24.13	NS
Heart weight (g)	0.930 ± 0.02	1.140 ± 0.22	0.012
Left ventricle weight (g)	0.760 ± 0.01	0.967 ± 0.07	0.015

SAP, systolic arterial pressure; DAP, diastolic arterial pressure; MAP, mean arterial pressure; LVEDP, left ventricular end-diastolic pressure, LVH, left ventricular hypertrophy; NS: not significant. Values are means ± S.E.M.

**Table 2:** **Echocardiographic characteristics in sham-operated and LV hypertrophy groups**.

	**Sham-operated (*n* = 10)**	**LVH (*n* = 10)**	***P***
LVFS (%)	41.93 ± 1.30	43.47 ± 1.93	NS
LVEF (%)	77.37 ± 1.50	78.95 ± 7.28	NS
LVEDD (mm)	7.94 ± 0.17	8.44 ± 0.27	NS
LVESD (mm)	4.61 ± 0.12	4.79 ± 0.30	NS
Sd (mm)	1.27 ± 0.03	1.42 ± 0.05	0.05
PWd (mm)	1.15 ± 0.03	1.40 ± 0.06	0.007
LVM (g)	1.07 ± 0.05	1.41 ± 0.10	0.02
IVRT (ms)	23.68 ± 0.12	23.83 ± 0.93	NS

LVFS, left ventricular fractional shortening; LVEF, left ventricular ejection fraction; LVEDD, left ventricular end-diastolic diameter; LVESD, left ventricular end-systolic diameter; Sd, septum wall thickness in diastole; PWd, posterior wall thickness in diastole; LVM, left ventricular mass; IVRT, isovolumic relaxation time. LVH, left ventricular hypertrophy; NS: not significant. Values are means ± S.E.M.

### Reduced mitochondrial respiratory chain complex activities in cardiac hypertrophy

Mitochondrial respiration measured *in situ* allows characterization of functional mitochondria in their normal intracellular assembly and position, preserving essential interactions with other organelles. Further, using different mitochondrial substrates and inhibitors, the relative contributions of respiratory chain complexes I, III, and IV to global mitochondrial respiratory rates can be determined.

**Figure 1:**
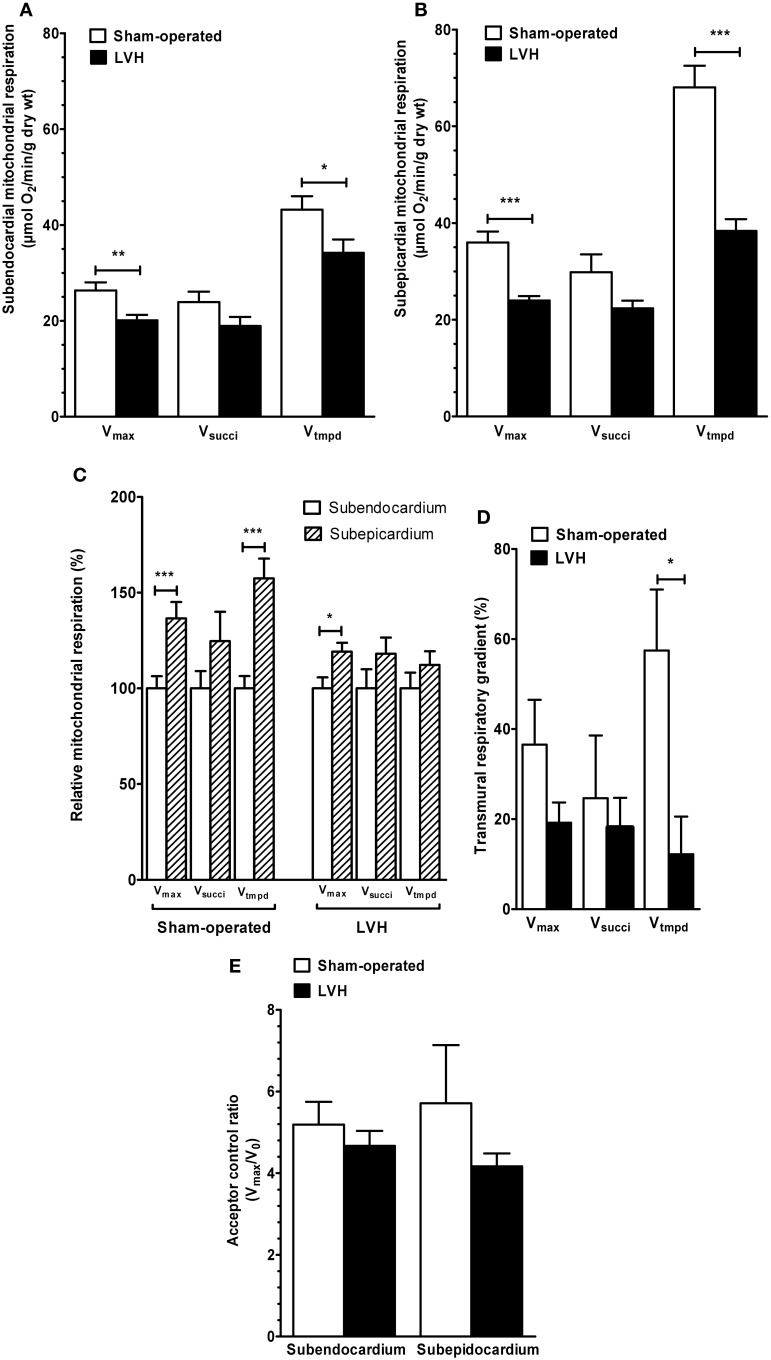
**Subendocardium and subepicardium LV mitochondrial respiratory chain complex activities and acceptor control ratio in sham-operated and LVH groups. (A)** Subendocardium mitochondrial respiratory chain complex activities. **(B)** Subepicardium mitochondrial respiratory chain complex activities. **(C)** Relative mitochondrial respiration in subendocardium and subepicardium, with the subendocardium layer considered as the reference (100%). **(D)** Transmural differences in mitochondrial respiratory chain complex activities across normal and hypertrophied left ventricles. **(E)** Subendocardium and subepicardium mitochondrial acceptor control ratio. *V*_max_, complexes I, III, and IV activities; *V*_succi_, complexes II, III, and IV activities; *V*_tmpd_, complex IV activity; Acceptor control ratio (*V*_max_/*V*_0_); *n* = 10 in sham-operated and left ventricular hypertrophy (LVH) groups. Values are means ± S.E.M. ^*^, *P* < 0.05; ^**^, *P* < 0.01; ^***^, *P* < 0.001.

*Concerning the endocardium*, maximal oxidative capacity (*V*_max_; complexes I, III, and IV activities) was significantly decreased in the LVH group (*n* = 10) compared with the sham-operated group (*n* = 10) (20.1 ± 1.1 vs. 26.3 ± 1.6 μmol O_2_/min/g dry weight, respectively; −23.6 %, *P* < 0.01; Figure 1A).

In the LVH group, *V*_succi_, (complexes II, III, and IV activities) tended to be decreased (18.9 ± 1.8 vs. 23.9 ± 2.1 μmol O_2_/min/g dry weight, respectively; −20.9%, *P* not significant).

*V*_tmpd_ (complex IV activity) was significantly decreased (34.1 ± 2.8 vs. 43.2 ± 2.7 μmol O_2_/min/g dry weight, respectively; −20.8%, *P* = 0.04).

*Concerning the epicardium*, similarly, *V*_max_ was significantly decreased in the LVH group compared with the sham-operated group (23.9 ± 0.9 vs. 35.9 ± 2.2 μmol O_2_/min/g dry weight, respectively; −33.3%, *P* < 0.001; Figure 1B). *V*_succi_ tended to be lower (22.3 ± 1.6 vs. 29.8 ± 3.6 μmol O_2_/min/g dry weight, respectively; −25.1%, *P* = 0.07). *V*_tmpd_ was significantly decreased (38.3 ± 2.4 vs. 68.0 ± 4.4 μmol O_2_/min/g dry weight, respectively, −43.6%, *P* < 0.001).

### Transmural differences in mitochondrial respiratory chain complex activities across the normal left ventricle and in LVH

To compare both LV layers, weI considered Epi mitochondrial complex activities normalized to their respective Endo values (Figure 1C).

In normal hearts, *V*_max_ was 36.5% lower (*P* < 0.01), *V*_succi_ was 24.6% lower (*P* not significant, 0.147) and *V*_tmpd_ was 57.4% lower (*P* < 0.05) in the Endo than in the Epi layers. Thus, normal LV is characterized by transmural differences in mitochondrial respiratory chain complexes I, III, and IV activities.

In the LVH group, *V*_max_ was 19.1% lower in Endo compared with Epi (*p* = 0.028), but *V*_succi_ and *V*_tmpd_ decrease (−18.0 and −12.2%, respectively) failed to reached statistical significance.

The ACR (*V*_max_/*V*_0_), representing the degree of coupling between oxidation and phosphorylation, was not different when comparing Epi and Endo LV layers both in sham and LVH rats (Figure 1D).

**Figure 2:**
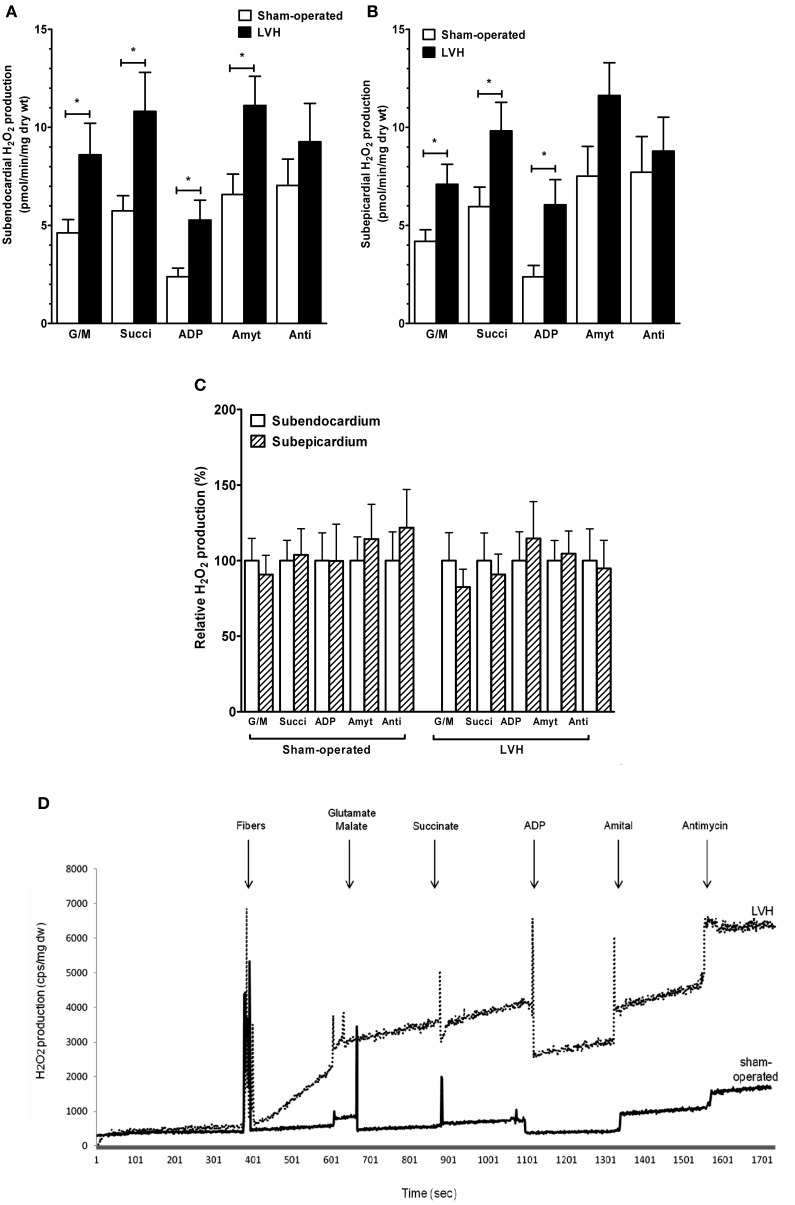
**Subendocardium and subepicardium hydrogen peroxide production in sham-operated and LV hypertrophy groups. (A)** Mitochondrial H_2_O_2_ production in subendocardium. **(B)** Mitochondrial H_2_O_2_ production in subepicardium. **(C)** Relative H_2_O_2_ production. **(D)** Representative trace of subendocardium H_2_O_2_ production in sham-operated and LV hypertrophy groups. Mitochondrial substrates were sequentially added. G/M, glutamate and malate are complex I substrates; Succi, succinate is complex II substrate; ADP, adenosine diphosphate is ATP synthase substrate; Amyt, amytal is a complex I inhibitor and Anti, antimycin A is a complex III inhibitor. Values are means ± S.E.M. ^*^, *P* < 0.05.

To determine whether pressure-overload modify the transmural differences in mitochondrial respiratory chain complex activities observed across the normal left ventricle, we compared such transmural gradient using the three main substrates. In the LVH group, the transmural respiratory gradient was significantly lower -as compared to normal LV- for *V*_tmpd_ assessing complex IV activity (−57.4 vs −12.2% for; *P* = 0.02, Figure 2). *V*_max_ and *V*_succi_ transmural energetic gradient were not significantly reduced in the LVH group (*P* = 0.18 and *P* = 0.95, respectively).

### Enhanced production of reactive oxygen species in subendocardium and subepicardium layers during cardiac hypertrophy

H_2_O_2_ production rate was calculated from the slope of ΔF/min, after subtracting background, from a standard curve established with the appropriate reaction conditions. At the conclusion of each experiment, fiber bundles were dried at 150°C, 15 min. H_2_O_2_ production is expressed as picomoles per minute per milligram of dry weight.

The relative contributions of respiratory chain complexes to global mitochondrial H_2_O_2_ production rate were determined using different substrates and inhibitors as depicted in the Figure legend.

The *subendocardial mitochondrial H_2_O_2_ production* in the presence of the complex I substrates glutamate and malate (Figure 2A) was significantly increased in the LVH group (*n* = 10) compared with the sham-operated animals (*n* = 10) (47.3 ± 8.7 vs. 25.4 ± 3.7 pmol/min/mg dry weight, respectively; +86.9%, *P* = 0.03).

In the presence of the complex II substrate succinate, H_2_O_2_ production was also higher in the LVH group than in the sham-operated group (59.4 ± 10.9 vs. 31.5 ± 4.24 pmol/min/mg dry weight, respectively; +89.4%, *P* = 0.04). In the presence of ADP, the H_2_O_2_ release was greater in the LVH group than in the sham-operated group (29.0 ± 5.5 vs. 13.1 ± 2.4 pmol/min/mg dry weight, respectively; +126.0%, *P* = 0.01). In the presence of amytal, a complex I inhibitor, the H_2_O_2_ release was higher in the LVH group than in the sham-operated group (61.1 ± 8.1 vs. 36.1 ± 5.7 pmol/min/mg dry weight, respectively; +70.7%, *P* = 0.02). Finally, in the presence of the complex III inhibitor antimycin A, the H_2_O_2_ release tended to be greater in the LVH group.

The *subepicardial H_2_O_2_ mitochondrial production* in the presence of glutamate and malate (Figure 2B) was significantly increased in the LVH group compared with the sham-operated group (39.0 ± 5.6 vs. 23.0 ± 3.2 pmol/min/mg dry weight, respectively; +73.1%, *P* = 0.02). With succinate as the substrate, H_2_O_2_ production was also higher in the LVH group than in the sham-operated group (53.9 ± 8.0 vs. 32.7 ± 5.4 pmol/min/mg dry weight, respectively; +66.1%, *P* = 0.02). The H_2_O_2_ release in the presence of ADP was also greater in the LVH group (33.3 ± 7.0 vs. 13.0 ± 3.1 pmol/min/mg dry weight, respectively; +163.0%, *P* = 0.01). In the presence of amytal and antimycin A, the H_2_O_2_ release did not differ significantly between the two groups.

The endocardial and epicardial LV production of H_2_O_2_ were similar in normal and in hypertrophied hearts, and no transmural gradient was observed with any substrate in either group (Figure 2C).

Figure 2D shows a representative trace of H_2_O_2_ production in Endo sham-operated and Endo LV hypertrophy groups.

Mitochondrial substrates were sequentially added. G/M, glutamate (5 mM) and malate (2 mM) are complex I substrates; Succi, succinate (5 mM) is complex II substrate; ADP, adenosine diphosphate (2 mM) is ATP synthase substrate; Amyt, amytal (2 mM) is a complex I inhibitor and Anti, antimycin A (8 microM) is a complex III inhibitor.

### Enhanced free radical leak in subendocardium and subepicardium layers during cardiac hypertrophy

To further analyze the production of ROS in Endo and Epi layers, we determined the mitochondrial H_2_O_2_ emission *per* O_2_ flux corresponding to the mitochondrial free radical leak (Picard et al., 2011).

**Figure 3:**
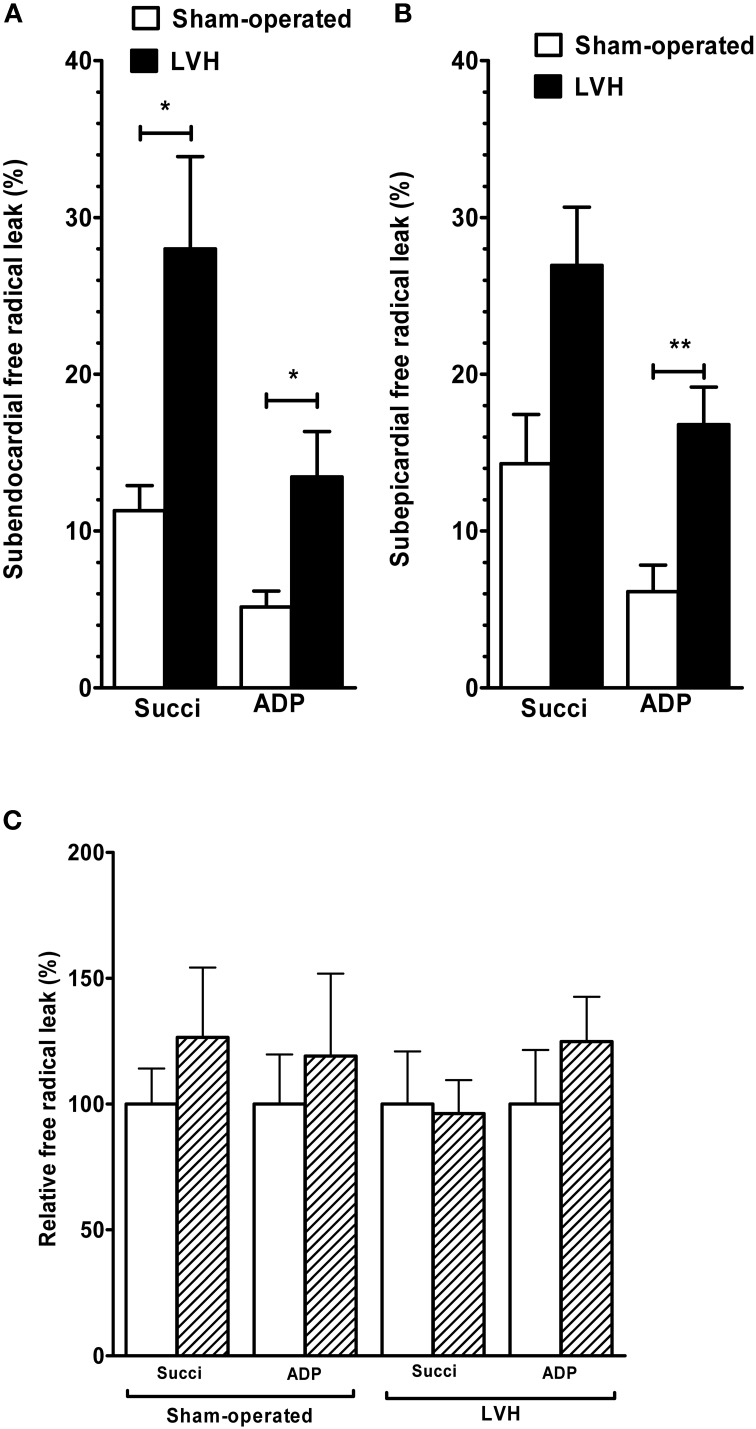
**Free radical leak in sham-operated and LV hypertrophy groups. (A)** Free radical leak in subendocardium. **(B)** Free radical leak in subepicardium. **(C)** Relative free radical leak in subendocardium and subepicardium, with the subendocardium layer considered as the reference (100%). The free radical leak represents the fraction of electrons which reduce O_2_ to ROS in the respiratory chain (H_2_O_2_ emission *per* O_2_ flux, expressed in %). Values are means ± S.E.M. ^*^, *P* < 0.05; ^**^, *P* < 0.01.

The *Endo mitochondrial free radical leak* in the presence of succinate (Figure 3A) was significantly increased in the LVH group compared with the sham-operated animals (28.0 ± 15.5 vs. 11.2 ± 4.2 %, respectively; +150.0%, *P* = 0.03). In the presence of ADP, free radical leak was also higher in the LVH group than in the sham-operated group (13.4 ± 2.8 vs. 5.1 ± 2.6%, respectively; +162.7%, *P* = 0.01).

The *Epi free radical leak* in the presence of succinate (Figure 3B) was not significantly different in the LVH group compared with the sham-operated group (26.9 ± 9.8 vs. 14.3 ± 8.2%, respectively; +88.1%, *P* = 0.07). The free radical leak in the presence of ADP was also greater in the LVH group (16.7 ± 6.3 vs. 6.1 ± 4.4%, respectively; +173.7%, *P* = 0.004).

The endocardial and epicardial LV free radical leak were similar in normal and in hypertrophied hearts, and no transmural gradient was observed with any substrate in either group (Figure 3C).

### Increased subendocardial and subepicardial mitochondrial content in cardiac hypertrophy

**Figure 4:**
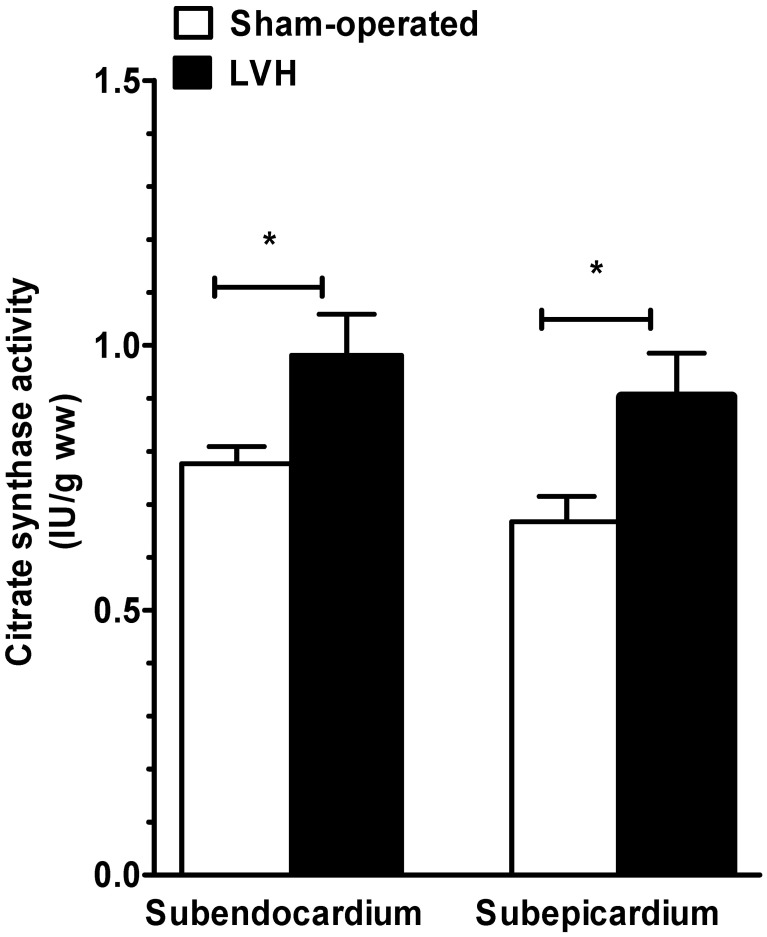
**Subendocardium and subepicardium citrate synthase activity in sham-operated and LVH groups.** Citrate synthase activity is used to approach global mitochondrial content in tissues and is expressed as units of activity per gram of tissue wet weight (IU.g ww).

We evaluated the global mitochondrial content in tissues by measuring the citrate synthase activity. During mild cardiac hypertrophy, mitochondrial content increased by 27.2 and 36.3% in Endo and Epi, respectively (Figure 4). No differences in citrate synthase activity through the different layers of the LV wall were observed either in the sham-operated or the LVH group.

**Figure 5:**
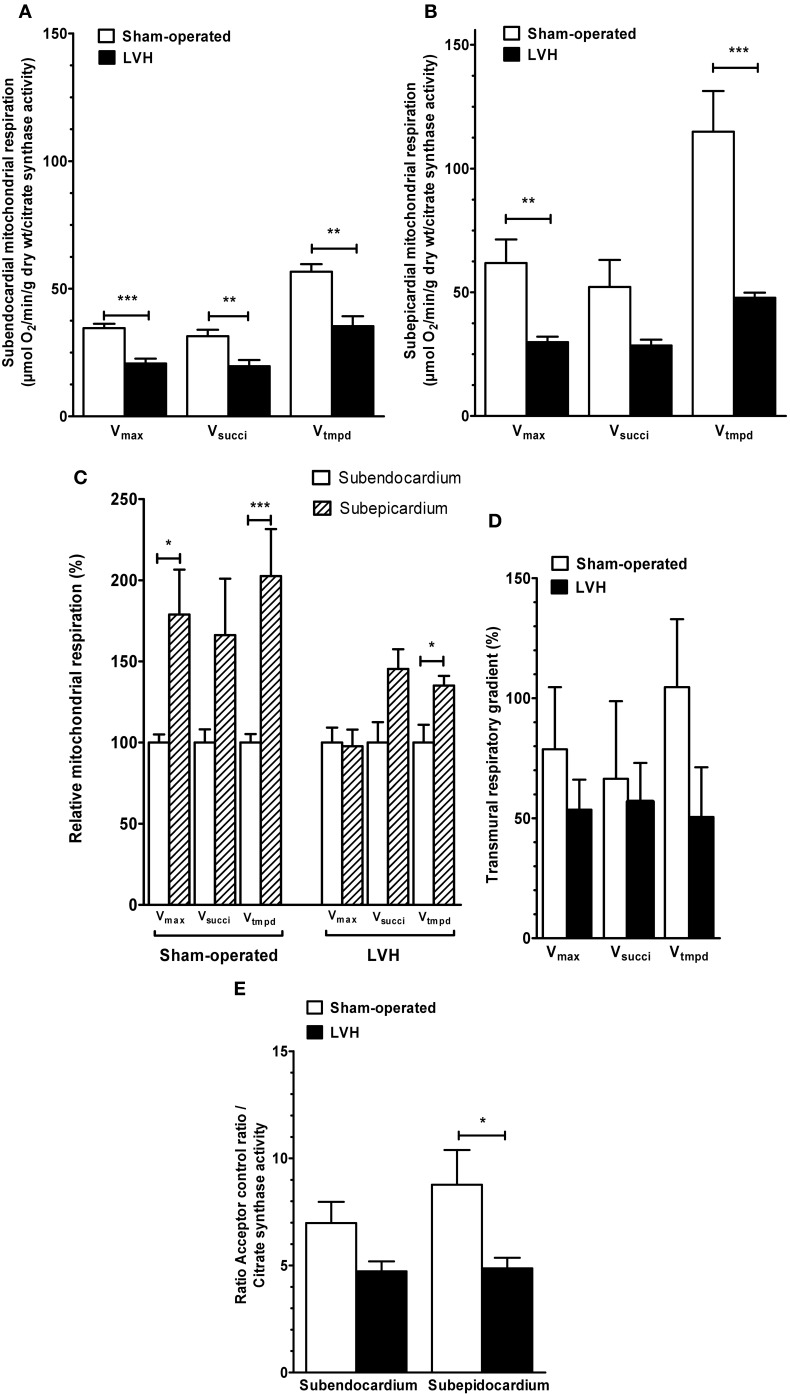
**Mitochondrial respiratory chain complex activities and acceptor control ratio in Sham-operated and LVH groups, normalized on citrate synthase activity. (A)** Normalized subendocardium mitochondrial respiratory chain complex activities. **(B)** Normalized subepicardium mitochondrial respiratory chain complex activities. **(C)** Normalized relative mitochondrial respiration in subendocardium and subepicardium, with the subendocardium layer considered as the reference (100%). **(D)** Normalized transmural differences in mitochondrial respiratory chain complex activities across normal and hypertrophied left ventricles. **(E)** Normalized acceptor control ratio. *V*_max_, complexes I, III, and IV activities; *V*_succi_, complexes II, III, and IV activities; *V*_tmpd_, complex IV activity; Acceptor control ratio (*V*_max_/*V*_0_); *n* = 10 in sham-operated and left ventricular hypertrophy (LVH) groups. Values are means ± S.E.M. ^*^, *P* < 0.05; ^**^, *P* < 0.01; ^***^, *P* < 0.001.

**Figure 6:**
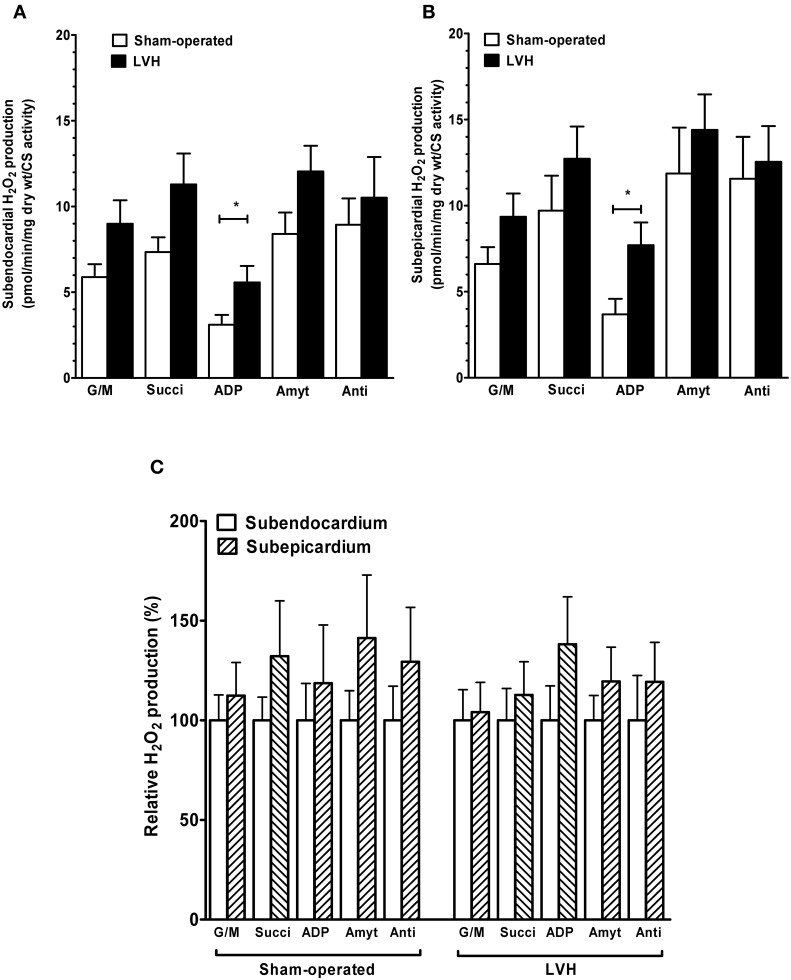
**Hydrogen peroxide production in sham-operated and LV hypertrophy groups, normalized on citrate synthase activity. (A)** Normalized mitochondrial H_2_O_2_ production in subendocardium. **(B)** Normalized mitochondrial H_2_O_2_ production in subepicardium. **(C)** Normalized relative H_2_O_2_ production. Mitochondrial substrates were sequentially added. G/M, glutamate and malate are complex I substrates; Succi, succinate is complex II substrate; ADP, adenosine diphosphate is ATP synthase substrate; Amyt, amytal is a complex I inhibitor and Anti, antimycin A is a complex III inhibitor. Values are means ± S.E.M. ^*^, *P* < 0.05.

When normalizing the data on CS activity, differences were amplified for respiration and reduced for H_2_O_2_ (Figures 5 and 6).

Thus, Endo and Epi mitochondrial respiratory chain complexes activities were globally decreased in LVH as compared to sham hearts (−40.0 %, *p* = 0.0006; −37.5 %, *p* = 0.0093; and −37.7 %, *p* = 0.0012 for *V*_max_, *V*_succi_ and *V*_tmpd_ in Endo and −51.7 %, *p* = 0.0041; −45.4 %, *p* = 0.073 and −58.4 %, *p* = 0.0006 for *V*_max_, *V*_succi_, and *V*_tmpd_ in Epi, respectively).

The relative mitochondrial respiration was increased in Epi, as compared to Endo, for *V*_max_ and *V*_tmpd_ in sham (+78.9 %, *p* = 0.026 and +102.6 %, *p* = 0.0006, respectively and for *V*_tmpd_ in LVH (+35.1 %, *p* = 0.0093) but statistical significance of complex 4 transmural gradient decrease in LVH was lost after normalization. Finally, ACR decrease was significant in LVH Epi (−44.49 %, *p* = 0.011).

Concerning H_2_O_2_ production, after normalizing on citrate synthase activity, LVH Endo and Epi still demonstrated significant increases as compared to sham hearts when using ADP as substrate (+79.4 %, *p* = 0.022 and +109.1 %, *p* = 0.027) in Endo and Epi, respectively. Again, endo and Epi relative H_2_O_2_ production did not differ in sham and in LVH groups.

## Discussion

The present study demonstrates (1) transmural differences in mitochondrial respiratory chain complex activities across the normal left ventricle, with a lower oxidative capacity of the Endo as compared to the Epi; (2) that aortic-banding-induced mild cardiac hypertrophy decreased mitochondrial oxidative capacities and enhanced hydrogen peroxide production; and (3) that cardiac hypertrophy was associated with a decreased transmural gradient in mitochondrial respiratory chain complex IV activity.

### Transmural differences in mitochondrial respiratory chain complex activities across the normal left ventricle

A considerable body of evidence has been accumulated regarding myocardial heterogeneity of the normal heart, including different blood flow, structure, metabolism, electrophysiological, and contractile properties and generally supporting increased mechanical stress and contractility in Endo (Whitty et al., 1976; Smith et al., 1990; Sonntag et al., 1996; Cazorla et al., 2005; Sengupta et al., 2006; Buckberg et al., 2008; Duncker and Bache, 2008; Lou et al., 2011; Van der Velden et al., 2011).

However, few data exist concerning the existence of an energetic gradient across the LV wall. Previous studies using isolated mitochondria showed either similar oxidative capacity in Endo and Epi (Whitty et al., 1976) or increased capacity in Endo (Camici et al., 1984). Respiration studies using isolated mitochondria might results in conflicting results, particularly in the case of small differences, because of non-homogeneity and fragility of mitochondria submitted to isolation procedures (Sharov et al., 1998). Such limitation might potentially be overcome by using saponin-skinned fibers that ensure global mitochondrial function assessment in intact mitochondria. MacDonald et al. recently investigated transmural differences in respiratory capacity across LV and no transmural respirational difference was observed in healthy rats (MacDonald et al., 2011). These data are in opposition with our results. Similar techniques and animals were used in both studies and further investigations will be useful to clearly explain such a discrepancy.

Indeed, we clearly observed that subendocardial oxidative capacity was significantly lower than subepicardial capacity. These differences hold true for complexes I, III, and IV of the mitochondrial respiratory chain. A key factor that might have been involved in our results is increased ROS production in Endo. Indeed, high oxidative stress has been related to mitochondrial dysfunction in several setting including ischemia-reperfusion and PO (Ingwall, 2009; Charles et al., 2011). Nevertheless, H_2_O_2_ overproduction in Endo was not observed, the relative H_2_O_2_ production being similar in both LV layers. Similar data were observed in LVH, further supporting that hydrogen peroxide did not explain the transmural gradient seen in mitochondrial respiration.

### Mild cardiac hypertrophy impairs subendocardial and subepicardial mitochondrial respiratory chain complex activities

Surprisingly, increased maximal oxidative capacity was transiently observed using isolated mitochondria in a thoracic aortic banding model. In this study, maximal respiratory capacity was greater than control in the first 6 weeks, normal after 10 weeks and significantly reduced after 20 weeks (Doenst et al., 2010). This might correspond to a first compensated phase, mitochondrial dysfunction occurring thereafter when ejection fraction was decreasing. Accordingly, it is generally accepted that mitochondrial dysfunction is observed at the stage of heart failure (Ventura-Clapier et al., 2001). In particular, energetic impairment has been reported when LVH is associated with cardiac dysfunction (Ventura-Clapier et al., 2001; Ali et al., 2011). A decreased rate of substrate oxidation predicts the onset of contractile dysfunction and heart failure in rats with PO (Neubauer, 2007; Jameel and Zhang, 2009; Doenst et al., 2010). However, whether mitochondrial dysfunction might be differently involved in endocardium as compared to epicardium during PO-induced mild cardiac hypertrophy development deserve further studies.

In the present work, for the first time, we observed that mitochondrial respiratory chain complex activities are significantly reduced in both the subendocardial and subepicardial LV layers in compensated LVH. This might be clinically relevant since our model of 6 weeks abdominal aortic banding induced an increase in LV mass of 31.7% with no clinical signs of heart failure and no changes in LVEDP, LV diameter, LV ejection fraction, LV fractional shortening, or IVRT. Furthermore, we used saponin-permeabilized fibers, which allow the determination of global mitochondrial function with respect to the mitochondrial architectural environment (Zoll et al., 2002). Similarly, MacDonald et al. also recently reported an early impairment in mitochondrial respiration in steptozotocin-induced diabetes (MacDonald et al., 2011).

Although mitochondrial uncoupling should not always be seen as deleterious (Duteil et al., 2010; Tran et al., 2012), besides mitochondrial respiratory chain activities impairment, PO might be associated with mitochondrial uncoupling particularly in case of heart failure. Accordingly, Faerber's et al. observed not only reduced respiratory rates but also reduced mitochondrial coupling in mice hearts after aortic banding performed between the brachiocephalic trunk and the left carotid artery (Faerber et al., 2011). Thus, a loss of efficiency in ATP production was observed both in the failing hearts and in cardiac hypertrophy without heart failure. In our study, at a global muscle level, we did not observe mitochondrial uncoupling. The smaller degree of LVH in our rats likely explains their maintained mitochondrial coupling.

### Mild cardiac hypertrophy is associated with increased mitochondrial hydrogen peroxide production and free radical leak

Excessive ROS generation likely participates in cardiac hypertrophy-associated mitochondrial damage (Pimentel et al., 2001; Seddon et al., 2007; Takimoto and Kass, 2007), and the sources of ROS production during cardiac hypertrophy deserve further investigation. Uncoupled nitric oxide synthetase, xanthine oxidoreductase, and NADPH oxidase might be important mediators of PO-induced LVH. However, a role of mitochondria in generating ROS in the setting of cardiac hypertrophy is also likely (Seddon et al., 2007). Indeed, the role of mitochondria extends far beyond energy production because mitochondria are important generators of ROS that can act as a source of cellular damage depending on the amount of ROS produced (Bouitbir et al., 2011).

Mitochondria are both causes and targets of ROS, and mitochondrial production of H_2_O_2_ might be increased as a result of LVH-associated mitochondrial dysfunction. The use of saponin-permeabilized fibers allowed us to study H_2_O_2_ production by mitochondria. The data thus obtained support the idea that mitochondria participate significantly in hypertrophy-related oxidative stress. Indeed, subendocardial and subepicardial H_2_O_2_ production was significantly enhanced in LVH compared to normal hearts. Accordingly, the free radical leak increased in LVH, supporting that the fraction of electrons which reduce O_2_ to ROS in the respiratory chain (Anderson et al., 2009; Bouitbir et al., 2012) was greater in pressure-overloaded hearts. This was observed in Endo and Epi and thus mitochondria located in both LV layers participated in the ROS overproduction of the hypertrophied hearts.

### Mild cardiac hypertrophy is associated with a decreased transmural gradient in mitochondrial respiratory chain complex IV activity

Interestingly, transmural complex IV activity was significantly reduced in LVH as compared to sham animals. Cardiac hypertrophy reduced such LV transmural gradient mainly through a decrease in subepicardial mitochondrial respiration.

A similar result has been reported during dobutamine-induced demand ischemia. Indeed, Jameel et al. observed in a stenotic perfused coronary bed during dobutamine-induced high cardiac work state, that the Epi showed greater metabolic changes than the Endo layer (Jameel et al., 2008). Although both settings were different, these two results might suggest that the sensitivity of the LV Epi layer might be increased in case of pre-existing pathology as compared to normal LV submitted to recent injury.

Whether mitochondrial dysfunction is not a major causative factor in the eventual failure of the LV cannot be inferred from our data since our model was not characterized by heart failure. Nevertheless, one might propose that the relatively light degree of LVH explains that only complex IV activity of the mitochondrial respiratory chain was significantly reduced as compared to sham heart. Further studies would be useful to determine whether greater LVH with beginning cardiac failure will be associated with a reduced LV transmural differences in all mitochondrial respiratory chain complexes activities. In this case, such a reduced transmural mitochondrial respiratory gradient might be a new marker of the transition from uncomplicated to complicated LVH.

### Increased subendocardial and subepicardial mitochondrial content in cardiac hypertrophy

It has been generally reported a reduced mitochondrial biogenesis in case of LVH, but mainly in case of decompensated pathological hypertrophy. Accordingly, Abel and Doenst recently proposed that mitochondrial biogenesis might be enhanced or maintained during physiological or compensated pathological hypertrophy, respectively (Abel and Doenst, 2011). In our study, the global mitochondrial content in tissues increased but mitochondrial oxidative capacity decreased concomitantly. Thus, as expected, our model likely allowed to explore LVH located just before a decompensated state.

Data can be normalized on CS in an attempt to view the results not as a whole but as data per unit of mitochondria. However, in this study, we performed citrate synthase analysis as additional measurements. Fibers were not the same than those used for mitochondrial respiration or H_2_O_2_ production, possibly decreasing the pertinence of such analysis. Accordingly, study normalizing respiration on citrate synthase used frozen myofibers bundles from respiratory assays (Picard et al., 2011). Despite this technical limitation, to discuss normalized data on CS activity appears interesting. Considering both normal heart and LVH, the data obtained without normalization are globally confirmed. Thus, differences were amplified for respiration and reduced for H_2_O_2_. To overcome the limitation related to the use of different fibers for H_2_O_2_ production and CS measurements, we determined the free radical leak, allowing determine mitochondrial ROS production on same fibers, matching respiration values with matched substrates. Such analysis confirmed that ROS originating from mitochondria were increased in LVH.

Concerning a potential transmural gradient in ROS production in normal heart, all analysis demonstrated no difference between Endo and Epi layers. This held true in LVH and supports that hydrogen peroxide does not explain the normal transmural gradient seen in respiration. Concerning the complex IV activity transmural gradient difference between normal heart and LVH, the statistical significance was lost when normalizing it on CS activity. This suggest caution in the interpretation of the data, as presented below.

## Limitations of the study

In case of obesity, an increased effect of blood pressure has been observed on LV growth resulting in a steeper slope of the blood pressure-LV mass index relation (Norton et al., 2009). Impaired pathways controlling cellular growth and proliferation appeared to be related to increased oxidative stress (Mandavia et al., 2012) and dietary-induced obesity has been shown to hasten the progression from concentric LV hypertrophy to pump dysfunction in SHR rats (but not in WKY control rats), independently of blood pressure changes (Majane et al., 2009). Since our animal weights were located at the upper limit of the normal range, it is possible that pressure-overload effects might have been enhanced as compared to animals with lower body weight.

Only part of the transmural difference in mitochondrial respiratory chain complex activities seen across the normal left ventricle was decreased in LVH. Since the statistical significance was lost when normalizing on citrate synthase activity, it might be viewed as no physiologically important. We believe that this is likely to be due to the relatively small degree of LVH in our experimental model and that even a small transmural difference might be pertinent. Indeed, keeping on mitochondrial function, for instance, very slight mitochondrial uncoupling of about 12% has been shown to be beneficial, protecting mice against decreased muscle oxidative capacities induced by sedentariness, development of type 2 diabetes and against high-caloric diet induced obesity (Duteil et al., 2010).

In summary, our data support a transmural difference in mitochondrial respiratory chain complex activities across the normal left ventricle, with a lower oxidative capacity of the Endo as compared to the Epi.

PO-induced mild cardiac hypertrophy was characterized by reduced mitochondrial respiratory chain complex activities associated with increased hydrogen peroxide production.

Additionally, cardiac hypertrophy might be associated with a decreased transmural gradient in mitochondrial respiratory chain complex IV activity. To determine whether these changes may be a hallmark in LVH time course needs further work aiming to investigate whether maladaptive hypertrophy is associated with a loss of LV transmural energetic gradient.

### Conflict of interest statement

The authors declare that the research was conducted in the absence of any commercial or financial relationships that could be construed as a potential conflict of interest.

## Abbreviations

Endo: subendocardium
Epi: subepicardium
LV: left ventricular
LVH: left ventricular hypertrophy
PO: pressure overload
ROS: reactive oxygen species
H_2_O_2_: hydrogen peroxide.

## References

[B1] Abel E. D. Doenst T. (2011). Mitochondrial adaptations to physiological vs. pathological cardiac hypertrophy. Cardiovasc. Res. 90, 234–342 10.1093/cvr/cvr015 21257612 PMC3115280

[B2] Ali A. Patel A. Ali Z. Abu-Omar Y. Saeed A. Athanasiou T. Pepper J. (2011). Enhanced left ventricular mass regression after aortic valve replacement in patients with aortic stenosis is associated with improved long-term survival. J. Thorac. Cardiovasc. Surg. 142, 285–291 10.1016/j.jtcvs.2010.08.084 21272899

[B3] Anderson E. J. Kypson A. P. Rodriguez E. Anderson C. A. Lehr E. J. Neufer P. D. (2009). Substrate-specific derangements in mitochondrial metabolism and redox balance in the atrium of the type 2 diabetic human heart. J. Am. Coll. Cardiol. 54, 1891–1898 10.1016/j.jacc.2009.07.031 19892241 PMC2800130

[B4] Anderson E. J. Neufer P. D. (2006). Type II skeletal myofibers possess unique properties that potentiate mitochondrial H(2)O(2) generation. Am. J. Physiol. Cell Physiol. 290, C844–C851 10.1152/ajpcell.00402.2005 16251473

[B5] Bouitbir J. Charles A. L. Echaniz-Laguna A. Kindo M. Daussin F. Auwerx J. Piquard F. Geny B. Zoll J. (2011). Opposite effects of statins on mitochondria of cardiac and skeletal muscles: a ‘mitohormesis’ mechanism involving reactive oxygen species and PGC-1. Eur. Heart J. 33, 1397–1407 10.1093/eurheartj/ehr224 21775390 PMC3365271

[B6] Bouitbir J. Daussin F. Charles A. L. Rasseneur L. Dufour S. Richard R. Piquard F. Geny B. Zoll J. (2012). Mitochondria of trained skeletal muscle are protected from deleterious effects of statins. Muscle Nerve. 10.1002/mus.23309 22907227

[B7] Buckberg G. Hoffman J. I. Mahajan A. Saleh S. Coghlan C. (2008). Cardiac mechanics revisited: the relationship of cardiac architecture to ventricular function. Circulation 118, 2571–2587 10.1161/CIRCULATIONAHA.107.754424 19064692

[B8] Camici P. Ursini F. Galiazzo F. Bellitto L. Pelosi G. Marzilli M. L'Abbate A. Barsacchi R. (1984). Different respiratory activities of mitochondria isolated from the subendocardium and subepicardium of the canine heart. Basic Res. Cardiol. 79, 454–460 6487238 10.1007/BF01908146

[B9] Cantor E. J. Babick A. P. Vasanji Z. Dhalla N. S. Netticadan T. (2005). A comparative serial echocardiographic analysis of cardiac structure and function in rats subjected to pressure or volume overload. J. Mol. Cell. Cardiol. 38, 777–786 10.1016/j.yjmcc.2005.02.012 15850571

[B10] Cazorla O. Szilagyi S. Le Guennec J. Y. Vassort G. Lacampagne A. (2005). Transmural stretch-dependent regulation of contractile properties in rat heart and its alteration after myocardial infarction. FASEB J. 19, 88–90 10.1096/fj.04-2066fje 15498894

[B11] Charles A. L. Guilbert A. S. Bouitbir J. Goette-Di Marco P. Enache I. Zoll J. Piquard F. Geny B. (2011). Effect of postconditioning on mitochondrial dysfunction in experimental aortic cross-clamping. Br. J. Surg. 24, 511–516 10.1002/bjs.7384 21259232

[B12] Doenst T. Pytel G. Schrepper A. Amorim P. Färber G. Shingu Y. Mohr F. W. Schwarzer M. (2010). Decreased rates of substrate oxidation *ex vivo* predict the onset of heart failure and contractile dysfunction in rats with pressure overload. Cardiovasc. Res. 86, 461–470 10.1093/cvr/cvp414 20035032

[B13] Drazner M. H. (2011). The progression of hypertensive heart disease. Circulation 123, 327–334 10.1161/CIRCULATIONAHA.108.845792 21263005

[B14] Duncker D. J. Bache R. J. (2008). Regulation of coronary blood flow during exercise. Physiol. Rev. 88, 1009–1086 10.1152/physrev.00045.2006 18626066

[B15] Duteil D. Chambon C. Ali F. Malivindi R. Zoll J. Kato S. Geny B. Chambon P. Metzger D. (2010). The transcriptional coregulators TIF2 and SRC-1 regulate energy homeostasis by modulating mitochondrial respiration in skeletal muscles. Cell Metab. 12, 496–508 10.1016/j.cmet.2010.09.016 21035760 PMC3032428

[B16] Faerber G. Barreto-Perreia F. Schoepe M. Gilsbach R. Schrepper A. Schwarzer M. Mohr F. W. Hein L. Doenst T. (2011). Induction of heart failure by minimally invasive aortic constriction in mice: reduced peroxisome proliferator-activated receptor γ coactivator levels and mitochondrial dysfunction. J. Thorac. Cardiovasc. Surg. 141, 492–500 10.1016/j.jtcvs.2010.03.029 20447656

[B17] Gallego-Delgado J. Lazaro A. Osende J. I. Barderas M. G. Duran M. C. Vivanco F. Egido J. (2006). Comparison of the protein profile of established and regressed hypertension-induced left ventricular hypertrophy. J. Proteome Res. 5, 404–413 10.1021/pr0503275 16457607

[B18] Geshi E. Konno N. Yanagishita T. Katagiri T. (1988). Impairment of mitochondrial respiratory activity in the early ischemic myocardium–with special reference to electron transport system. Jpn. Circ. J. 52, 535–542 2845164 10.1253/jcj.52.535

[B19] Griffiths E. R. Friehs I. Scherr E. Poutias D. McGowan F. X. Del Nido P. J. (2010). Electron transport chain dysfunction in neonatal pressure-overload hypertrophy precedes cardiomyocyte apoptosis independent of oxidative stress. J. Thorac. Cardiovasc. Surg. 139, 1609–1617 10.1016/j.jtcvs.2009.08.060 20038480 PMC2875266

[B20] Ingwall J. S. (2009). Energy metabolism in heart failure and remodelling. Cardiovasc. Res. 81, 412–419 10.1093/cvr/cvn301 18987051 PMC2639129

[B21] Jameel M. N. Wang X. Eijgelshoven M. H. Mansoor A. Zhang J. (2008). Transmural distribution of metabolic abnormalities and glycolytic activity during dobutamine-induced demand ischemia. Am. J. Physiol. Heart Circ. Physiol. 6, H2680–H2686 10.1152/ajpheart.01383.2007 18424629 PMC2614109

[B22] Jameel M. N. Zhang J. (2009). Myocardial energetics in left ventricular hypertrophy. Curr. Cardiol. Rev. 5, 243–250 10.2174/157340309788970379 20676284 PMC2822148

[B23] Kuznetsov A. V. Veksler V. Gellerich F. N. Saks V. Margreiter R. Kunz W. S. (2008). Analysis of mitochondrial function *in situ* in permeabilized muscle fibers, tissues and cells. Nat. Protoc. 3, 965–976 10.1038/nprot.2008.61 18536644

[B24] Lou Q. Fedorov V. V. Glukhov A. V. Moazami N. Fast V. G. Efimov I. R. (2011). Transmural heterogeneity and remodeling of ventricular excitation-contraction coupling in human heart failure. Circulation 123, 1881–1890 10.1161/CIRCULATIONAHA.110.989707 21502574 PMC3100201

[B25] MacDonald J. R. Oellermann M. Rynbeck S. Chang G. Ruggiero K. Cooper G. J. Hickey A. J. (2011). Transmural differences in respiratory capacity across the rat left ventricle in health, aging, and streptozotocin-induced diabetes mellitus: evidence that mitochondrial dysfunction begins in the subepicardium. Am. J. Physiol. Cell Physiol. 300, C246–C255 10.1152/ajpcell.00294.2010 21084644

[B26] Majane O. H. Vengethasamy L. du Toit E. F. Makaula S. Woodiwiss A. J. Norton G. R. (2009). Dietary-induced obesity hastens the progression from concentric cardiac hypertrophy to pump dysfunction in spontaneously hypertensive rats. Hypertension 54, 1376–1383 10.1161/HYPERTENSIONAHA.108.127514 19841294

[B27] Mandavia C. H. Pulakat L. Demarco V. Sowers J. R. (2012). Over-nutrition and metabolic cardiomyopathy. Metab. Clin. Exp. 10.1016/j.metabol.2012.02.013 22465089 PMC3393834

[B28] Murdoch C. E. Zhang M. Cave A. C. Shah A. M. (2006). NADPH oxidase-dependent redox signalling in cardiac hypertrophy, remodelling and failure. Cardiovasc. Res. 71, 208–215 10.1016/j.cardiores.2006.03.016 16631149

[B29] Nabeebaccus A. Zhang M. Shah M. A. (2011). NADPH oxidase and cardiac remodeling. Heart Fail. Rev. 16, 5–12 10.1007/s10741-010-9186-2 20658317

[B30] Neubauer S. (2007). The failing heart–an engine out of fuel. N. Engl. J. Med. 356, 1140–1151 10.1056/NEJMra063052 17360992

[B31] Norton G. R. Majane O. H. Libhaber E. Maseko M. J. Makaula S. Libhaber C. Woodiwiss A. J. (2009). The relationship between blood pressure and left ventricular mass index depends on an excess adiposity. J. Hypertens. 27, 1873–1883 10.1097/HJH.0b013e32832dca53 19512944

[B32] Opie L. H. Commerford P. J. Gersh B. J. Pfeffer M. A. (2006). Controversies in ventricular remodelling. Lancet 367, 356–367 10.1016/S0140-6736(06)68074-4 16443044

[B33] Picard M. Ritchie D. Thomas M. M. Wright K. J. Hepple R. T. (2011). Alterations in intrinsic mitochondrial function with aging are fiber type-specific and do not explain differential atrophy between muscles. Aging Cell 10, 1047–1055 10.1111/j.1474-9726.2011.00745.x 21933339

[B34] Pimentel D. R. Amin J. K. Xiao L. Miller T. Viereck J. Oliver-Krasinski J. Baliga R. Wang J. Siwik D. A. Singh K. Pagano P. Colucci W. S. Sawyer D. B. (2001). Reactive oxygen species mediate amplitude-dependent hypertrophic and apoptotic responses to mechanical stretch in cardiac myocytes. Circ. Res. 89, 453–460 11532907 10.1161/hh1701.096615

[B35] Rimbaud S. Sanchez H. Garnier A. Fortin D. Bigard X. Veksler V. Ventura-Clapier R. (2009). Stimulus specific changes of energy metabolism in hypertrophied heart. J. Mol. Cell. Cardiol. 46, 952–959 19452634 10.1016/j.yjmcc.2009.01.013

[B36] Seddon M. Looi Y. H. Shah A. M. (2007). Oxidative stress and redox signalling in cardiac hypertrophy and heart failure. Heart 93, 903–907 10.1136/hrt.2005.068270 16670100 PMC1994416

[B37] Sengupta P. P. Korinek J. Belohlavek M. Narula J. Vannan M. A. Jahangir A. Khandheria B. K. (2006). Left ventricular structure and function: basic science for cardiac imaging. J. Am. Coll. Cardiol. 48, 1988–2001 10.1016/j.jacc.2006.08.030 17112989

[B38] Sharov V. G. Goussev A. Lesch M. Goldstein S. Sabbah H. N. (1998). Abnormal mitochondrial function in myocardium of dogs with chronic heart failure. J. Mol. Cell. Cardiol. 30, 1757–1762 10.1006/jmcc.1998.0739 9769231

[B39] Smith S. H. Kramer M. F. Reis I. Bishop S. P. Ingwall J. S. (1990). Regional changes in creatine kinase and myocyte size in hypertensive and nonhypertensive cardiac hypertrophy. Circ. Res. 67, 1334–1344 2147129 10.1161/01.res.67.6.1334

[B40] Sonntag M. Deussen A. Schultz J. Loncar R. Hort W. Schrader J. (1996). Spatial heterogeneity of blood flow in the dog heart. I. Glucose uptake, free adenosine and oxidative/glycolytic enzyme activity. Pflugers Arch. 432, 439–450 10.1007/s004240050156 8766004

[B41] Srere P. A. (1969). Citrate synthase. Meth. Enzymol. 13, 3–11

[B42] Takimoto E. Kass D. A. (2007). Role of oxidative stress in cardiac hypertrophy and remodeling. Hypertension 49, 241–248 10.1161/01.HYP.0000254415.31362.a7 17190878

[B43] Tran T. N. Noll E. Collange O. Bouitbir J. Charles A. L. Kindo M. Zoll J. Diemunsch P. Geny B. (2012). Mitochondrial respiratory chain uncoupling, oxidative stress and skeletal muscle energetics. Skeletal Muscle Physiol. Classif. Dis.

[B44] Van Bilsen M. Van Nieuwenhoven F. A. Van der Vusse G. J. (2009). Metabolic remodelling of the failing heart: beneficial or detrimental? Cardiovasc. Res. 81, 420–428 10.1093/cvr/cvn282 18854380

[B45] Van der Velden J. Merkus D. de Beer V. Hamdani N. Linke W. A. Boontje N. M. Stienen G. J. Duncker D. J. (2011). Transmural heterogeneity of myofilament function and sarcomeric protein phosphorylation in remodeled myocardium of pigs with a recent myocardial infarction. Front. Physiol. 2:83 10.3389/fphys.2011.00083 22131977 PMC3223384

[B46] Veal E. A. Day A. M. Morgan B. A. (2007). Hydrogen peroxide sensing and signalling. Mol. Cell 26, 1–14 10.1016/j.molcel.2007.03.016 17434122

[B47] Veksler V. I. Kuznetsov A. V. Sharov V. G. Kapelko V. I. Saks V. A. (1987). Mitochondrial respiratory parameters in cardiac tissue: a novel method of assessment by using saponin-skinned fibers. Biochim. Biophys. Acta 892, 191–196 10.1016/0005-2728(87)90174-5 3593705

[B48] Ventura-Clapier R. Garnier A. Veksler V. Joubert F. (2001). Bioenergetics of the failing heart. Biochim. Biophys. Acta 1813, 1360–1372 10.1016/j.bbamcr.2010.09.006 20869993

[B49] Whitty A. J. Dimino M. J. Elfont E. A. Hughes G. W. Repeck M. W. (1976). Transmural mitochondrial differences in myocardium. Recent Adv. Stud. Cardiac Struct. Metab. 11, 349–354 1031934

[B50] Zoll J. Monassier L. Garnier A. N'Guessan B. Mettauer B. Veksler V. Piquard F. Ventura-Clapier R. Geny B. (2006). ACE inhibition prevents myocardial infarction-induced skeletal muscle mitochondrial dysfunction. J. Appl. Physiol. 101, 385–391 10.1152/japplphysiol.01486.2005 16614354

[B51] Zoll J. Ponsot E. Doutreleau S. Mettauer B. Piquard F. Mazzucotelli J. P. Diemunsch P. Geny B. (2005). Acute myocardial ischemia induces specific alterations of ventricular mitochondrial function in experimental pigs. Acta Physiol. Scand. 185, 25–32 10.1111/j.1365-201X.2005.01458.x 16128694

[B52] Zoll J. Sanchez H. N'Guessan B. Ribera F. Lampert E. Bigard X. Serrurier B. Fortin D. Geny B. Veksler V. Ventura-Clapier R. Mettauer B. (2002). Physical activity changes the regulation of mitochondrial respiration in human skeletal muscle. J. Physiol. 543, 191–200 10.1113/jphysiol.2002.019661 12181291 PMC2290497

